# Prenatal Exposure to Environmentally-Relevant Contaminants Perturbs Male Reproductive Parameters Across Multiple Generations that are Partially Protected by Folic Acid Supplementation

**DOI:** 10.1038/s41598-019-50060-z

**Published:** 2019-09-25

**Authors:** Maryse Lessard, Pauline M. Herst, Phanie L. Charest, Pauline Navarro, Charles Joly-Beauparlant, Arnaud Droit, Sarah Kimmins, Jacquetta Trasler, Marie-Odile Benoit-Biancamano, Amanda J. MacFarlane, Mathieu Dalvai, Janice L. Bailey

**Affiliations:** 1 Department of Animal Sciences, Faculty of Agricultural and Food Sciences, Centre de recherche en reproduction, développement et santé intergénérationnelle, Laval University, Quebec City, Quebec Canada; 2 Department of Nutrition, Faculty of Agricultural and Food Sciences, Centre de recherche en reproduction, développement et santé intergénérationnelle, Laval University, Quebec City, Quebec Canada; 3 Computational Biology Laboratory Research Centre, Faculty of Medicine, Laval University, Quebec City, Quebec Canada; 4 Department of Pharmacology and Therapeutics, Faculty of Medicine, McGill University, Montreal, Quebec Canada; 5 Department of Animal Science, Faculty of Agricultural and Environmental Sciences, McGill University, Montreal, Quebec Canada; 6 Departments of Pediatrics, Human Genetics and Pharmacology & Therapeutics, McGill University, Research Institute, McGill University Health Centre, Montreal, Quebec Canada; 7 Department of Pathology and Microbiology, Faculty of Veterinary Medicine, University of Montreal, Saint-Hyacinthe, Quebec Canada; 8 Nutrition Research Division, Health Canada, Ottawa, Canada

**Keywords:** Nutritional supplements, Spermatogenesis

## Abstract

The paternal environment is thought to influence sperm quality and future progeny may also be impacted. We hypothesized that prenatal exposure to environmentally-relevant contaminants impairs male reproduction, altering embryo gene expression over multiple generations. Folic acid (FA) can improve sperm quality and pregnancy outcomes, thus we further hypothesized that FA mitigates the contaminants. Sprague-Dawley F0 female rats treated with persistent organic pollutants (POPs) or corn oil and fed basal or supplemented FA diets, then used to yield four generations of litters. Only F0 females received POPs and/or FA treatments. In utero POPs exposure altered sperm parameters in F1, which were partly rescued by FA supplementation. Paternal exposure to POPs reduced sperm quality in F2 males, and the fertility of F3 males was modified by both POPs and FA. Ancestral FA supplementation improved sperm parameters of F4 males, while the POPs effect diminished. Intriguingly, F3 males had the poorest pregnancy outcomes and generated the embryos with the most significantly differentially expressed genes. Early-life exposure to POPs harms male reproduction across multiple generations. FA supplementation partly mitigated the impact of POPs. The two-cell embryo transcriptome is susceptible to paternal environment and could be the foundation for later pregnancy outcomes.

## Introduction

Levels of persistent organic pollutants (POPs), including polychlorinated biphenyls (PCBs) and legacy pesticides such as dichlorodiphenyltrichloroethane (DDT) have been declining since their use was restricted by the Stockholm Convention in 2001^1^. Nonetheless, due to their long half-life and lipophilic nature, they remain present in the environment^2^. Many POPs are known to be endocrine disruptors^3^ and may impair spermatogenesis and sperm function, thereby reducing male fertility and reproductive health^4,5^. With the alarming decline in sperm counts since 1970, it is of interest to determine whether and to what extent POPs may play in male fertility^6^.

In men, semen quality can be considered a biomarker for overall health^6–8^. Men with reduced sperm quality have a shorter life expectancy^8^ and have a higher risk of adverse health outcomes such as cardiovascular disease, hypertension and diabetes^6,7^. Exposure of men to POP contaminants may thus influence not just their fertility but also their overall health^9,10^.

There is an increasing body of research demonstrating that the adverse effects of environmental contaminants can be transmitted to subsequent generations^11–13^. Although historical presumption links the health of subsequent generations to the mother^14^, paternal exposures can also affect the development of his descendants^15,16^.

Maternal folic acid (FA) supplementation in the periconceptional period reduces neural tube defects^17,18^ and supplementation is recommended for women of childbearing age^19^. In Canada, and many other countries, higher folic acid intake is ensured by mandatory fortification of white wheat flour and other enriched grain products^20^ Folic acid deficiency in mouse models reduces sperm function and sperm count^21–23^ and supplementation could protect the father’s sperm from the adverse effects of environmental contaminants^24^.

This study tested the hypothesis that prenatal exposure to environmentally-relevant POPs disrupts sperm quality, fertility and early embryo gene expression across multiple, unexposed generations in a rat model. The POPs composition and dosage has been previously confirmed to be comparable to lower concentrations measured in maternal and umbilical cord plasma in Arctic populations^25^. Further, dietary FA supplementation was assessed for whether it mitigated the effects of POPs to improve male reproductive parameters.

## Results

### Sperm characteristics following exposure to POPs and/or FA

The impact of POPs and/or FA supplementation exposure on sperm characteristics (counts, morphology and viability) are presented in Table 1. Testicular spermatid count is lower in the F2 generation for rats sired by F1 fathers whose germ cells were directly exposed to POPs *in utero* through F0 females’ exposure (*p* = 0.01). The effect of FA supplementation on sperm morphology varied due to POPs in the F2 generation. When the F2 rats were not exposed to POPs, FA supplementation decreased normal sperm morphology. When combined with POPs, FA supplementation brings sperm morphology to levels comparable to control group (CTRL; *p* = 0.01). Sperm viability decreased due to POPs exposure in both F1 and F2 generations (*p* = 0.02; *p* = 0.003). FA-supplementation partially restored sperm viability in the F1 rats, but not in the F2 generation. (*p* = 0.23).

**Table 1:** Sperm characteristics.

(*n* = 12)	Testicular spermatid count (x10^6^ ± SEM)	*Cauda* epididymal sperm count (x10^6^ ± SEM)	Sperm morphology (% ± SEM)	Sperm viability (% ± SEM)
**F1**				
CTRL	74.32 ± 6.5	73.85 ± 3.8	92 ± 2	48^a^ ± 3
POPs	70.68 ± 6.1	70.44 ± 4.0	90 ± 2	34^b^ ± 3
FA	71.83 ± 6.1	74.91 ± 3.8	91 ± 2	45^a^ ± 3
POPsFA	69.50 ± 6.5	71.71 ± 4.0	88 ± 2	44^a^ ± 3
*p* value	POPs (*p* = 0.63)	POPs (*p* = 0.40)	POPs (*p* = 0.26)	POPs (*p* = 0.009)
	FA (*p* = 0.80)	FA (*p* = 0.77)	FA (*p* = 0.60)	FA (*p* = 0.17)
	POPs*FA (*p* = 0.91)	POPs*FA (*p* = 0.97)	POPs*FA (*p* = 0.88)	POPs*FA ( ***p*** ** = 0.02)**
**F2**				
CTRL	80.04^a^ ± 6.2	82.42 ± 5.8	96^a^ ± 1	39^a^ ± 2
POPs	63.60^b^ ± 5.6	65.14 ± 6.7	95^a^ ± 1	30^b^ ± 2
FA	78.40^a^ ± 6.2	76.43 ± 5.8	93^b^ ± 1	39^a^ ± 2
POPsFA	57.24^b^ ± 7.7	76.98 ± 6.0	96^a^ ± 1	35^b^ ± 2
*p* value	POPs (*p* = 0.01)	POPs (*p* = 0.17)	POPs (*p* = 0.18)	POPs (*p* = 0.003)
	FA (*p* = 0.64)	FA (*p* = 0.63)	FA (*p* = 0.09)	FA (*p* = 0.32)
	POPs*FA (*p* = 0.72)	POPs*FA (*p* = 0.14)	POPs*FA (*p* = 0.01)	POPs*FA (*p* = 0.23)
**F3**				
CTRL	66.39 ± 12.1	110.41 ± 10.9	92 ± 2	37^a^ ± 3
POPs	47.75 ± 12.6	103.77 ± 10.9	93 ± 2	29^a^ ± 3
FA	46.16 ± 10.6	109.48 ± 10.9	91 ± 2	39^b^ ± 3
POPsFA	57.11 ± 10.7	105.03 ± 10.9	91 ± 2	39^b^ ± 3
*p* value	POPs (*p* = 0.75)	POPs (*p* = 0.92)	POPs (*p* = 0.74)	POPs (*p* = 0.09)
	FA (*p* = 0.62)	FA (*p* = 0.98)	FA (*p* = 0.53)	**FA (*p*** = **0.03)**
	POPs*FA (*p* = 0.26)	POPs*FA (*p* = 0.61)	POPs*FA (*p* = 0.55)	POPs*FA (*p* = 0.14)
**F4**				
CTRL	71.85 ± 11.0	111.72 ± 9.0	94 ± 1	57 ± 3
POPs	62.67 ± 11.0	120.37 ± 9.0	92 ± 1	54 ± 3
FA	90.39 ± 11.0	120.29 ± 9.0	94 ± 1	53 ± 3
POPsFA	77.39 ± 10.9	135.26 ± 9.0	94 ± 1	54 ± 3
*p* value	POPs (*p* = 0.23)	POPs (*p* = 0.19)	POPs (*p* = 0.35)	POPs (*p* = 0.71)
	FA (*p* = 0.37)	FA (*p* = 0.19)	FA (*p* = 0.34)	FA (*p* = 0.42)
	POPs*FA (*p* = 0.88)	POPs*FA (*p* = 0.72)	POPs*FA (*p* = 0.38)	POPs*FA (*p* = 0.51)

Testicular spermatid counts in F1-F4 generation. F1 to F4 rat spermatozoa concentration (*cauda* epididymal sperm count normalized by epididymal weight). F1–F4 rat spermatozoa morphology. F1 to F4 rat spermatozoa viability. SEM = standard error of the mean.

Prenatal POPs exposure reduced sperm motility and progressive motility in the F1 males (*p* = 0.04; Table 2). Similarly, prenatal POPs exposure and FA supplementation separately decreased both the straightness coefficient (STR; *p* = 0.02) and the linearity coefficient (LIN; *p* = 0.01). When combined POPs and FA, the coefficient returned to a level similar to CTRL in the same generation. Interestingly, POPs did not alter sperm motility in the F2 generation, although FA supplementation decreased both STR (*p* = 0.02) and LIN (*p* = 0.03; Table 2).

**Table 2:** Sperm motility parameters in F1-F4 generations.

*(n* = *12)*	Total motility (%) ± SEM	Progressive motility (%) ± SEM	VAP (µm/s) ± SEM	VSL (µm/s) ± SEM	VCL (µm/s) ± SEM	ALH (µm/s) ± SEM	BCF (Hz) ± SEM	STR (%) ± SEM	LIN (%) ± SEM
**F1**									
CTRL	78^a^ ± 6	67^a^ ± 5	326.7 ± 18	243.6 ± 16	504.9 ± 23	20.1 ± 0.5	15.9 ± 0.9	67^a^ ± 1	46^a^ ± 1
POPs	64^b^ ± 6	53^b^ ± 5	282.9 ± 18	204.6 ± 16	454.6 ± 23	19.5 ± 0.5	17.7 ± 0.9	65^b^ ± 1	42^b^ ± 1
FA	82^a^ ± 6	70^a^ ± 5	316.0 ± 18	228.8 ± 16	503.3 ± 23	20.8 ± 0.5	17.2 ± 0.9	65^b^ ± 1	42^b^ ± 1
POPsFA	72^b^ ± 6	61^b^ ± 5	308.4 ± 18	227.4 ± 16	485.0 ± 23	20.0 ± 0.5	16.9 ± 0.9	66^a^ ± 1	45^a^ ± 1
*p* value	**POPs (*p*** = **0.04)**	**POPs (*p*** = **0.04)**	POPs (*p* = 0.17)	POPs (*p* = 0.20)	POPs (*p* = 0.14)	POPs (*p* = 0.14)	POPs (*p* = 0.39)	POPs (*p* = 0.69)	POPs (*p* = 0.44)
	FA (*p* = 0.32)	FA (*p* = 0.31)	FA (*p* = 0.69)	FA (*p* = 0.79)	FA (*p* = 0.53)	FA (*p* = 0.20)	FA (*p* = 0.77)	FA (*p* = 0.69)	FA (*p* = 0.87)
	POPs*FA (*p* = 0.74)	POPs*FA (*p* = 0.63)	POPs*FA (*p* = 0.33)	POPs*FA (*p* = 0.23)	POPs*FA (*p* = 0.49)	POPs*FA (*p* = 0.80)	POPs*FA (*p* = 0.26)	POPs*FA (*p* = **0.02)**	POPs*FA (*p* = **0.01)**
**F2**									
CTRL	52 ± 7	43 ± 6	212.5 ± 9	159.3 ± 8	319.6 ± 12	13.7 ± 0	14.9 ± 0.9	68^a^ ± 1	47^a^ ± 2
POPs	42 ± 8	32 ± 7	207.7 ± 11	164.4 ± 10	299.1 ± 13	12.5 ± 1	14.8 ± 1.0	70^a^ ± 1	50^a^ ± 2
FA	56 ± 7	42 ± 6	207.7 ± 9	151.0 ± 8	307.0 ± 12	13.3 ± 0	14.6 ± 0.9	66^b^ ± 1	44^b^ ± 2
POPsFA	53 ± 8	41 ± 6	189.1 ± 10	139.9 ± 9	286.7 ± 12	12.6 ± 1	15.7 ± 0.9	66^b^ ± 1	45^b^ ± 2
*p* value	POPs (*p* = 0.40)	POPs (*p* = 0.37)	POPs (*p* = 0.24)	POPs (*p* = 0.72)	POPs (*p* = 0.10)	POPs (*p* = 0.09)	POPs (*p* = 0.60)	POPs (*p* = 0.36)	POPs (*p* = 0.26)
	FA (*p* = 0.34)	FA (*p* = 0.50)	FA (*p* = 0.24)	FA (*p* = 0.06)	FA (*p* = 0.31)	FA (*p* = 0.80)	FA (*p* = 0.77)	**FA (*p*** = **0.02)**	**FA (*p*** = **0.03)**
	POPs*FA (*p* = 0.69)	POPs*FA (*p* = 0.44)	POPs*FA (*p* = 0.45)	POPs*FA (*p* = 0.36)	POPs*FA (*p* = 0.99)	POPs*FA (*p* = 0.67)	POPs*FA (*p* = 0.53)	POPs*FA (*p* = 0.44)	POPs*FA (*p* = 0.58)
**F3**									
CTRL	48 ± 7	37 ± 5	241.2 ± 10	173.7 ± 8	398.2 ± 17	18.6 ± 7	19.4 ± 0.7	64^a^ ± 1	41 ± 1
POPs	46 ± 7	34 ± 6	227.5 ± 10	151.0 ± 8	370.3 ± 17	18.0 ± 7	20.3 ± 0.7	61^b^ ± 1	40 ± 1
FA	44 ± 7	34 ± 6	237.7 ± 10	161.6 ± 8	387.8 ± 17	32.4 ± 7	20.1 ± 0.7	62^a^ ± 1	41 ± 1
POPsFA	52 ± 7	39 ± 6	242.2 ± 10	163.7 ± 8	397.5 ± 17	18.1 ± 7	20.4 ± 0.7	61^b^ ± 1	40 ± 1
*p* value	POPs (*p* = 0.66)	POPs (*p* = 0.79)	POPs (*p* = 0.65)	POPs (*p* = 0.21)	POPs (*p* = 0.58)	POPs (*p* = 0.29)	POPs (*p* = 0.41)	**POPs (*p*** = **0.02)**	POPs (*p* = 0.21)
	FA (*p* = 0.87)	FA (*p* = 0.88)	FA (*p* = 0.59)	FA (*p* = 0.96)	FA (*p* = 0.62)	FA (*p* = 0.33)	FA (*p* = 0.56)	FA (*p* = 0.80)	FA (*p* = 0.87)
	POPs*FA (*p* = 0.48)	POPs*FA (*p* = 0.43)	POPs*FA (*p* = 0.38)	POPs*FA (*p* = 0.13)	POPs*FA (*p* = 0.26)	POPs*FA (*p* = 0.33)	POPs*FA (*p* = 0.68)	POPs*FA (*p* = 0.26)	POPs*FA (*p* = 0.81)
**F4**									
CTRL	56 ± 6	43 ± 5	221.0 ± 21	146.6^a ± ^20	349.0^a^ ± 23	16.8 ± 1	18.9 ± 0.9	64 ± 1	43 ± 1
POPs	57 ± 6	45 ± 5	231.6 ± 21	167.1^a^ ± 20	350.2^a^ ± 23	15.7 ± 1	18.5 ± 0.9	66 ± 1	46 ± 1
FA	65 ± 6	52 ± 5	250.9 ± 21	184.7^b^ ± 20	385.6^b^ ± 23	16.9 ± 1	17.4 ± 0.9	66 ± 1	45 ± 1
POPsFA	58 ± 6	45 ± 5	279.9 ± 21	219.3^b^ ± 21	409.3^b^ ± 23	16.1 ± 1	17.5 ± 0.9	68 ± 1	47 ± 1
*p* value	POPs (*p* = 0.56)	POPs (*p* = 0.62)	POPs (*p* = 0.34)	POPs (*p* = 0.17)	POPs (*p* = 0.58)	POPs (*p* = 0.10)	POPs (*p* = 0.87)	POPs (*p* = 0.14)	POPs (*p* = 0.14)
	FA (*p* = 0.44)	FA (*p* = 0.38)	FA (*p* = 0.07)	**FA (*p*** = **0.03)**	**FA (*p*** = **0.05)**	FA (*p* = 0.62)	FA (*p* = 0.16)	FA (*p* = 0.08)	FA (*p* = 0.31)
	POPs*FA (*p* = 0.45)	POPs*FA (*p* = 0.35)	POPs*FA (*p* = 0.65)	POPs*FA (*p* = 0.72)	POPs*FA (*p* = 0.62)	POPs*FA (*p* = 0.83)	POPs*FA (*p* = 0.76)	POPs*FA (*p* = 0.91)	POPs*FA (*p* = 0.69)

(VAP: Average path velocity; VSL: Linear velocity; VCL: Curvilinear velocity; ALH: mean amplitude of lateral head displacement; BCF: frequency of head displacement; STR: straightness coefficient; LIN: linearity coefficient). SEM = standard error of the mean.

POPs exposure and/or FA supplementation also affected sperm in paternal lineages not directly exposed (F3 and F4 generations). Ancestral POPs treatment decreased STR (*p* = 0.02) in F3 males, while, the linear velocity (VSL; *p* = 0.03) and the curvilinear velocity (VCL; *p* = 0.05) parameters increased with ancestral FA supplementation in F4 males (Table 2). FA supplementation increased sperm viability in the F3 lineage (*p* = 0.03; Table 1).

### POPs and/or FA supplementation alter gene expression in two-cell embryos

**Figure 1:**
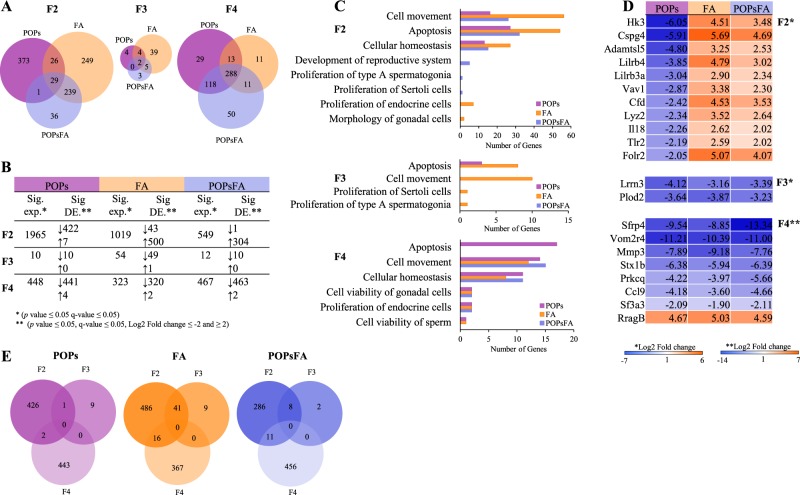
Differential gene expression in two-cell embryos due to prenatal paternal POPs, FA and POPsFA exposure. (**A**) Venn diagrams comparing the number of significantly differentially expressed genes due to POPs, FA and POPsFA in F2-F4 two-cell embryos. (**B**) Table including total number of significantly expressed (Sig. exp.) genes (*p* value ≤ 0.05, FDR ≤5%) and the number of significantly differentially expressed (Sig. DE) genes (*p* value ≤ 0.05, FDR ≤5% and −2≥ |Log2 Fold change| ≥2) that are up- or downregulated indicated by ↑ and ↓ respectively due to POPs, FA and POPsFA in F2-F4. (**C**) Gene ontology (GO) and pathway analysis showing only significantly affected pathways (*p* value ≤ 0.05). (**D**) Heatmaps representing common genes shared between all treatments per generation. (**E**) Candidate genes that are altered across multiple generations due to POPs, FA and POPsFA across F2-F4.

To understand whether the sperm from exposed males induced a phenotype in subsequent generations, we examined the transcriptome of F2 two-cell embryos (Fig. 1). The two-cell embryo stage was specifically chosen, because the gene expression patterns are more related to the paternal genome^26,27^.

First, we analyzed the impact of each treatment on gene expression and overlap of significantly differentially expressed (DE) genes (*p* value ≤ 0.05, FDR ≤5% and −2 ≥ |Log2 Fold change| ≥2) between treatments compared to CTRL in F2-F4 (Fig. 1A). In F2, 29 significantly DE genes were common among POPs, FA and POPsFA treatments (Supplementary Table 1). POPs and POPsFA shared only one common gene in F2; though, FA and POPsFA, and POPs and FA share 239 and 26 significantly DE genes, respectively (Fig. 1A). The number of significantly DE genes decreased for all treatments in F3 (POPs 429 to 10; FA 543 to 50; POPsFA 305 to 10); this dilution effect supports the rationale that reprogramming will minimize generational transmission. In comparison to the F2, F3 gene expression more closely resembles that of the unexposed CTRL. In F4, however, the number of significantly DE genes increased substantially POPs 10 to 445; FA 50 to 322 and POPsFA 10 to 465.

Next, we examined the distribution of up- and down-regulated genes due to POPs and/or FA supplementation compared to CTRL in F2-F4 (Fig. 1B). In F2, of the 429 POPs-induced significantly DE genes, 422 were down-regulated (|Log2 Fold change| ≤−2) and 7 were up-regulated (|Log2 Fold change| ≥2). In contrast, the majority of all significantly DE genes in FA and POPsFA embryos were upregulated, FA 43↓; 500↑ and POPsFA 1↓; 304↑, respectively. This expression pattern changed in F3 and F4 such that all treatments decreased gene expression (Fig. 1B).

We performed gene-ontology analysis (GO) to identify pathways altered by the significantly DE genes due to direct and ancestral exposure to POPs and/or FA supplementation in F2-F4 (Fig. 1C). In F2, exposure to POPs, FA and POPsFA affected multiple similar pathways (*p* ≤ 0.05) notably cell movement, apoptosis and cell homeostasis. Interestingly, pathways involved in male germ cell development were only affected by POPsFA in F2 (*p* ≤ 0.05). In contrast, in F3, those male germ cell development pathways were affected in FA embryos. In F4, multiple different pathways were altered by all treatment groups, except apoptosis, which was altered in POPs embryos.

To determine whether dietary FA supplementation rescued the impact of POPs on two-cell embryo transcriptomes, we compared the gene expression of shared genes among all treatments in F2-F4 (Fig. 1D). In F2, 29 significantly DE genes (*p* value ≤ 0.05, FDR ≤5% and −2≥ |Log2 Fold change| ≥2) were shared among POPs, FA and POPsFA, of which 27 genes were down-regulated in POPs embryos and up-regulated in FA embryos. The same 27 genes were up-regulated in POPsFA embryos but to a lesser extent. Only two genes, RragB and LOC108348078 (encodes ATP-dependent zinc metalloprotease YME1L1), were affected (up or down-regulated) by all treatments. This consistent effect was only observed in F2 and disappeared in subsequent generations (Fig. 1D).

Lastly, we identified genes that were significantly DE (*p* value ≤ 0.05, FDR ≤5% and −2≥ |Log2 Fold change| ≥2) across generations. Prenatal paternal POPs exposure led to one conserved significantly DE gene between F2 and F3. FA and POPsFA, however, altered 41 and 8 significantly DE genes from F2 to F3, respectively. None of these genes were significantly altered from F2-F4 in all treatments.

### Ancestral POPs and/or FA supplementation were associated with negative pregnancy outcomes

Direct exposure of POPs and/or FA supplementation had no apparent effect on fertility parameters of the F1 and F2 males or pregnancy outcomes in their litters. We observed, however, transgenerational effects on pregnancy outcomes (Table 3), as the number of fetuses sired by F3 fathers decreased with ancestral POPs exposure (*p* = 0.04). The harmful effect of ancestral POPs exposure was also apparent as the number of implantation sites tended to be lower than CTRL (*p* = 0.06).

**Table 3:** Pregnancy outcomes for F1-F3 fathers.

	Nb fetuses	Implantation sites	Fertility rate (% ± SEM)	Preimplantation losses (% ± SEM)	Postimplantation losses (% ± SEM)	Neonatal deaths (% ± SEM)
**F1**						
CTRL	12 ± 1	13 ± 1	85 ± 7	11 ± 4	4 ± 8	18 ± 12
POPs	13 ± 1	14 ± 1	85 ± 7	10 ± 4	6 ± 7	20 ± 13
FA	14 ± 1	14 ± 1	90 ± 7	9 ± 4	1 ± 7	1 ± 13
POPsFA	11 ± 1	13 ± 1	82 ± 7	6 ± 4	16 ± 7	2 ± 12
*p* value	POPs (*p* = 0.38)	POPs (*p* = 0.66)	POPs (*p* = 0.62)	POPs (*p* = 0.64)	POPs (*p* = 0.31)	POPs (*p* = 0.92)
	FA (*p* = 0.88)	FA (*p* = 1.00)	FA (*p* = 0.90)	FA (*p* = 0.52)	FA (*p* = 0.67)	FA (*p* = 0.19)
	POPs*FA (*p* = 0.15)	POPs*FA (*p* = 0.20)	POPs*FA (*p* = 0.58)	POPs*FA (*p* = 0.77)	POPs*FA (*p* = 0.40)	POPs*FA (*p* = 0.95)
**F2**						
CTRL	12 ± 2	12 ± 2	85 ± 13	8 ± 13	7 ± 3	10 ± 5
POPs	14 ± 2	15 ± 2	89 ± 15	6 ± 15	4 ± 3	6 ± 5
FA	10 ± 2	10 ± 2	68 ± 13	30 ± 13	3 ± 3	2 ± 5
POPsFA	12 ± 2	12 ± 2	96 ± 15	1 ± 15	5 ± 3	1 ± 5
*p* value	POPs (*p* = 0.27)	POPs (*p* = 0.30)	POPs (*p* = 0.28)	POPs (*p* = 0.33)	POPs (*p* = 0.77)	POPs (*p* = 0.60)
	FA (*p* = 0.37)	FA (*p* = 0.30)	FA (*p* = 0.73)	FA (*p* = 0.58)	FA (*p* = 0.64)	FA (*p* = 0.19)
	POPs*FA (*p* = 0.99)	POPs*FA (*p* = 0.97)	POPs*FA (*p* = 0.41)	POPs*FA (*p* = 0.37)	POPs*FA (*p* = 0.44)	POPs*FA (*p* = 0.64)
**F3**						
CTRL	14^a^ ± 1	14^a^ ± 1	84^a^ ± 8	14^a^ ± 7	2^a ± ^6	11.3 ± 9
POPs	12^b^ ± 1	13^b^ ± 1	88^a^ ± 8	11^a^ ± 7	1^a^ ± 6	1.3 ± 8
FA	13^a^ ± 1	14^a^ ± 1	89^a^ ± 8	5^a^ ± 7	6^b^ ± 6	3.6 ± 9
POPsFA	9^b^ ± 1	11^b^ ± 1	54^b^ ± 8	33^b^ ± 7	24^b^ ± 6	15.6 ± 8
*p* value	**POPs (*p*** = **0.04)**	**POPs (*p*** = **0.06)**	POPs (*p* = 0.08)	POPs (*p* = 0.09)	POPs (*p* = 0.16)	POPs (*p* = 0.90)
	FA (*p* = 0.11)	FA (*p* = 0.43)	FA (*p* = 0.10)	FA (*p* = 0.35)	**FA (*p*** = **0.03)**	FA (*p* = 0.71)
	POPs*FA (*p* = 0.32)	POPs*FA (*p* = 0.61)	POPs*FA (*p* = **0.03)**	POPs*FA (*p* = **0.04)**	POPs*FA (*p* = 0.12)	POPs*FA (***p*** **=** 0.23)

Fertility rate corresponds to (# fetuses/# *corpus lutea*)*100, pre-implantation losses corresponds to ((# *corpus lutea* – # implantation sites)/# *corpus lutea*)*100 and post-implantation losses corresponds to ((# implantation sites - # fetuses)/# implantation sites)*100. Neonatal deaths correspond to pups dead after birth during the first week of life (PND 1 to PND 6).

FA supplementation did not protect against POPs in our study, since the fertility rate was lower (*p* = 0.03) and preimplantation losses are higher (*p* = 0.04) in litters sired by the F3 fathers who were ancestrally exposed to both POPs and FA supplementation (Table 3). Moreover, there were more postimplantation losses due to ancestral FA supplementation in the absence of POPs (*p* = 0.03). Fertility parameters were not studied in the fourth generation as the F4 males were not mated.

## Discussion

An increasing body of evidence suggests that, in men, poor sperm parameters are predictive of other pathologies later in life^6–8^. Therefore, our observations that direct and ancestral exposure to environmental contaminants decreases sperm function is of concern. We report here that prenatal exposure of the paternal lineage to a POPs mixture induces subtle reductions in sperm quality (F1-F4) and male fertility (F1-F3) in a Sprague-Dawley rat model, thereby affecting males that were not directly exposed to the contaminants. Concomitant supplementation with FA was hypothesized to counteract harmful effects of POPs, however, relatively moderate effects were observed, albeit over multiple generations.

These modest phenotypes likely reflect the physiologically appropriate treatments tested. Indeed, the present study used oral administration of environmentally relevant POPs concentrations intended to approximate body burdens in Inuit people^25^ as a pertinent model of contemporary pollutant exposure. In addition, we used a mixture of contaminants and the cumulative effect may differ from effects observed using a single contaminant^28^. Moreover, the dietary FA levels used are physiological, not pharmacological. The control diet represents recommended adult intake (0.4 mg/d) and the supplemented diet reflects the FA intake levels of women in Canada, consuming FA-fortified food and a daily periconceptional supplements, corresponding to 3-fold the recommended dietary allowance^29^.

### Generational adverse effects due to prenatal paternal POPs exposure

#### F1 generation – POPs particularly affected sperm viability and motility

Prenatal paternal POPs exposure caused moderate, but adverse effects on sperm parameters (F1-F4) and male fertility (F1-F3). Sperm viability and motility appeared to be the most sensitive to POPs exposure, impacting predominantly sperm from F1 and F2 males (Tables 1, 2). Similar effects were observed in men from the far North as they were exposed to POP contaminants, through their diet^30–32^. Other regions of concern include malaria-endemic regions, as previous studies indicate that men have lower sperm quality associated with DDT exposure^33,34^.

Several POP chemicals have structural similarities with naturally occurring steroid and thyroid hormones, which may induce pseudo-hormonal or endocrine-disrupting behaviors causing imbalances in normal physiological processes^35^. Multiple studies demonstrated a link between prenatal exposure to endocrine disruptors and altered male reproductive health in adulthood^36–38^. Endocrine disruption during a critical fetal developmental window may affect endocrine homeostatic mechanisms, thereby compromising adult reproductive function^39^ and explaining the reduced sperm quality observed in F1 rats following prenatal POPs exposure.

#### F2 generation – Prenatal POPs exposure altered F2 two-cell embryo gene expression and sperm phenotype in adult F2 males

Emerging evidence supports the concept that prenatal exposure to environmental pollutants can alter the sperm phenotype of subsequent generations^40^. To determine whether the altered sperm parameters in POPs-exposed F1 males compromised the F2 generation, we examined early-embryo gene expression and later, sperm parameters in the adult F2 males. The two-cell embryo stage was selected because paternal epigenetic reprogramming is not yet complete and allows observation of heritable paternal traits^27^.

Gene expression was dramatically down-regulated due to prenatal paternal POPs exposure in F2 two-cell embryos (Fig. 1B). Interestingly, gene ontology analysis revealed that similar pathways including cell movement, apoptosis and cellular homeostasis were affected by POPs (Fig. 1D). Since the F2 two-cell embryos were not directly exposed to the treatments, it can be assumed that differences in their gene expression profiles are due to *in utero* POPs exposure in F1 male germ cells.

Furthermore, these early embryo changes could explain the observed differences in sperm quality and overall phenotypic outcomes later in adult F2 males. The testicular spermatid counts (Table 1) and sperm viability (Table 1) were decreased due to POPs in F2 adults.

#### Ancestral POPs exposure affects particularly F4 two-cell embryo gene expression

The F3 generation is the first unexposed and any phenotypes observed considered to be transgenerational. Therefore, appearance of any phenotypic traits due to F1 exposure is solely dependent on transmission via the male germ cell lineage.

Ancestral exposure to POPs still perturbed gene expression in the F3 two-cell embryos, albeit less than in the F2 embryos (10 versus 429, respectively). The affected genes in the F3 embryos are implicated in apoptosis (Fig. 1C, F3 graph). Furthermore, only one gene, V-set immunoregulatory receptor (*Vsir*; ENSRNOG00000000569), was significantly DE expressed in both F2 and F3 two-cell embryos.

Perturbed gene expression in the embryos must be due to paternal factors in our model. Male germ cells undergo extensive epigenomic reprogramming during development from primordial germ cells in the fetal gonad through postnatal spermatogenesis and after fertilization, and are thus vulnerable to environmental stressors during these reprogramming windows^41^. Prenatal paternal POPs exposure may have altered epigenetic marks in the F1 sperm that are transmitted to the F2 two-cell embryos, thereby changing their gene expression^42^ and even postnatal development and health^43^.

Indeed, in F3 adult males, ancestral POPs exposure altered sperm function (Table 2), in concordance with a previous rat study where ancestral DDE diminished the percentage of motile sperm until F3^44^.

In the F4 two-cell embryos, 448 genes were significantly DE as a result of ancestral POPs exposure of the F3 males. The majority of these genes were down-regulated and again implicated in apoptosis, but also cell movement, cellular homeostasis, viability of gonadal cells, proliferation of endocrine cells and germ cell viability. There were no significantly DE genes common to all generations of embryos. It is tempting to speculate that POPs exposure of F1 germ cells induced some gene modifications that escape reprogramming and are responsible for the embryo and adult phenotypes observed in subsequent generations.

Nonetheless, ancestral POPs exposure did not affect F4 sperm quality parameters.

### Protective effect of FA supplementation

To the best of our knowledge, this is the first report that attempts to counter the harmful effects of POPs. We selected FA supplementation as a nutritional intervention, since it is well known to reduce the incidence of congenital birth anomalies^45,46^ and to improve sperm quality in men^47^. Furthermore, dietary FA has been shown to counteract the effects of bisphenol A following maternal exposure^48^ and protected against chromatin damage and mutation in the male germline^29^.

In support of our hypothesis, FA supplementation protected F1 sperm viability (Table 1) and motility parameters (Table 2) against prenatal POPs exposure. Recent studies have also shown FA to protect sperm quality against lead^49^ and the carbamate insecticide, methyomyl^50^. Furthermore, a high dose of FA (20 mg/kg/day) protected male germ cells, including sperm viability against the harmful effects of bisphenol A in a rat model^51^. It should be noted, however, that similar levels of FA are reported to induce adverse health outcomes^52,53^.

Partially supporting our hypothesis, in the F2 two-cell embryos, prenatal paternal exposure to FA supplementation with POPs counteracted (upregulated) the POPs-induced downregulation of shared genes (Fig. 1D). Interestingly, pathways implicated in sperm development were among those that were only affected by prenatal paternal FA supplementation with POPs, although whether these are beneficial is unknown according to our gene ontology analysis (Fig. 1C). In F2 adults, POPs and FA interact, suggesting that FA supplementation is beneficial for sperm morphology in the presence of these contaminants (Table 1). In contrast, when F2 males were only exposed to FA supplementation, the percentage of morphologically normal sperm (Table 1) and motility parameters dropped (Table 2). Relevant to this F2 sperm phenotype, prenatal paternal exposure to FA supplementation alone markedly altered gene pathways related to cell movement, gonadal cell morphology and apoptosis in the F2 embryos (Fig. 1C). Corresponding to these pathways, previous studies have shown that moderate to high (2–10X) gestational FA supplementation is linked to altered DNA methylation patterns in offspring somatic tissues, including the sperm and brain^53–55^.

Similar to what was observed in the F3 POPs two-cell embryos, a small number of genes were significantly DE in POPsFA embryos, and the two shared genes were downregulated as a result of both treatments, which does not support our hypothesis (Fig. 1A,B). Unlike the F2 embryos, therefore, FA supplementation did not counteract the POPs effects in the F3 embryos (Fig. 1D). With respect to sperm, no phenotypic alterations were observed in F3 POPsFA males, although ancestral FA supplementation in the absence of POPs increased sperm viability (Table 1).

In the F4 embryos, the number of significantly DE genes increased substantially compared to the F3 embryos due to all lineage treatments. In contrast to our hypothesis, genes were expressed always in the same manner across treatments, thus ancestral FA supplementation did not counter the POPs. Even several generations after initial exposure, FA supplementation increased sperm motility parameters, VSL and VCL, in the F4 male lines.

### Pregnancy outcomes are affected in F3

To our knowledge, we are the first to show the transgenerational effect of an environmentally relevant pollutant mixture at a physiological dose that extends to the F4 generation. Previous studies have demonstrated that exposure to POPs has little to no immediate effect on pregnancy outcomes^56,57^. In contrast, our study demonstrates that the F3 paternal lineage showed the worst pregnancy outcomes amongst all generations (Table 3). These results are unexpected, especially since the F3 generation did not display any altered sperm parameters, however, transcriptomic analysis of the F4 two-cell embryos revealed a surprisingly high number of significantly DE genes with all treatments (Fig. 1A). These early developmental alterations could be the foundation for later adverse pregnancy phenotypes (Table 3).

Although we have no mechanistic evidence for these observations, we postulate that the sperm produced by the F3 paternal lineage contained accumulated upstream epimutations such as perturbed DNA methylation, histone modification, or noncoding RNAs, that escaped remodeling during development and spermatogenesis. These epimutations in the F3 sperm were unable to be corrected, resulting in substantial alternations in the F4 two-cell embryo transcriptome and associated pregnancy outcomes. Thus, the F3 males did not appear to have sperm parameter abnormalities, yet their reproductive competence was compromised. Also, although not assessed in this study, DNA sequence changes cannot be not ruled out^58^.

Various indicators of pregnancy outcome from both the F3 POPs and POPsFA lineages indicate compromised paternal competence to produce healthy litters (Table 3). Therefore, no counteracting effect of FA supplementation against POPs was observed in F4 pregnancy outcomes; in fact, the FA supplemented lineage also showed increased postimplantation fetal losses (Table 3).

## Conclusion

Semen quality should be now considered as a biomarker of overall health^6,8^, since men with reduced sperm quality also have a shorter life expectancy and a higher risk of adverse health outcomes such as cardiovascular disease, hypertension and diabetes^7^. The present study confirms our hypothesis that prenatal exposure to environmentally-relevant POPs subtly but significantly reduces male reproductive function for at least three generations. These results with a rodent model support speculation that declining sperm quality in men may be due to environmental factors^15,35,59,60^. In contrast, our results do not fully support the hypothesis that prenatal FA supplementation offsets the effects of POPs. Compared to other animal studies using non-physiological FA doses, our FA supplementation was modest, which may explain the lack of convincing results in its ability to protect paternal lineages from prenatal exposure to POPs. This finding, however, does not negate the public health benefit of periconceptional FA supplementation in reducing neural tube defects^46,61,62^.

## Materials and Methods

### Environmentally-relevant mixture

As described previously, the POPs mixture^25,63^ was designed to approximate the profile the Canadian northern food chain^64^. The experimental dose, which is considered to be environmentally-relevant^25^, is reported as 500 µg PCBs/kg body weight plus other POPs in lower proportions (Table 4).

**Table 4:** Composition of POPs mixture used^25,63^.

Products	CAS no.	Origin^a^	% in mixture	Dose (µg/kg body weight)
Aroclor and congener neat mix^b^		AccuStandard	32.4	500
Technical chlordane	57-74-9	AccuStandard	21.4	330.3
Dichlorodiphenyldichloroethylene *(p,p*′-DDE)	72-55-9	Sigma-Aldrich	19.3	297.8
Dichlorodiphenyltrichloroethane (*p,p*′-DDT)	50-29-3	SigmaAldrich	6.8	104.9
Technical toxaphene	8001-35-2	AccuStandard	6.5	100.0
*α-*hexachlorocyclohexane (α-HCH)	319-84-6	Sigma-Aldrich	6.2	95.7
Aldrin	309-00-2	Sigma-Aldrich	2.5	38.6
Dieldrin	60-57-1	Sigma-Aldrich	2.1	32.4
1,2,4,5-tetrachlorobenzene	95-94-3	Sigma-Aldrich	0.9	13.9
Dichlorodiphenyldichloroethane (*p, p*′-DDD)	72-54-8	Sigma-Aldrich	0.5	7.7
*β-*hexachlorocyclohexane (*β-*HCH)	319-85-7	Sigma-Aldrich	0.4	6.2
Hexachlorobenzene	118-74-1	AccuStandard	0.4	6.2
Mirex	2385-85-5	Sigma-Aldrich	0.2	3.1
Lindane	58-89-9	Sigma-Aldrich	0.2	3.1
Pentachlorobenzene	608-93-5	Sigma-Aldrich	0.2	3.1

^a^AccuStandard Inc (New Haven, Connecticut); Sigma-Aldrich Inc (St Louis, Missouri).

^b^Mix containing PCBs: Aroclor 1260 (58.9%); Aroclor 1254 (39.3%); 2,4,4′-trichlorobiphenyl (PCB 28; 1%); 2,2′,4,4′-tetrachlorobiphenyl (PCB 47; 0.8%); 3,3′,4,4′,5-pentachlorobiphenyl (PCB 126; 0.02%), and 3,3′,4,4′-tetrachlorobiphenyl (PCB 77; 0.004%).

### Diets

Animals were fed one of two FA-defined diets based on the AIN-93G formula^65^: control (2 mg FA/kg diet) or supplemented (6 mg FA/kg diet) (# 110700 and #117819 respectively by Dyets, Inc, Bethlehem, PA). The control diet approximates the FA intake of Canadian adults during the post-fortification era (0.4 mg/day), whereas the supplemented diet corresponds to the total FA intake from fortified foods plus a daily multivitamin containing 1 mg FA^22^.

### Study design

Animal care and all treatment procedures were in accordance with the guidelines of the Canadian Council on Animal Care and approved by the Université Laval animal Research Ethics Committee (certificate # 2015010-2). Forty healthy five-week-old female Sprague-Dawley rats (Charles River Laboratories, Saint-Constant, Quebec, Canada) were used as founder dams (F0). Rats were housed in standard cages at 22 °C (50% humidity) with 12-hour light-dark cycle. Food and water were provided *ad libitum*.

**Figure 2:**
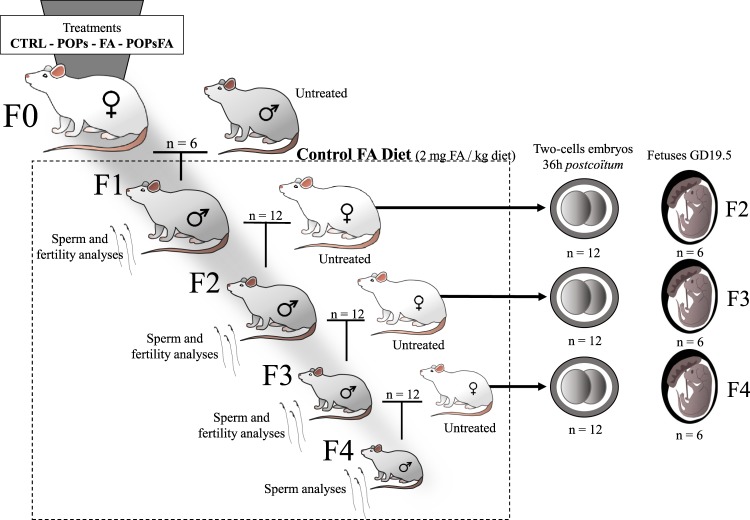
Study design. Four treatment groups of Sprague-Dawley F0 founder females (n = 6) were gavaged with an environmentally-relevant POPs mixture or corn oil and received either a control diet (2 mg FA/kg diet) or supplemented diet (6 mg FA/kg diet). F0 females were treated for 5 weeks and then mated to untreated males; POPs and FA supplementation treatments continued until the birth of the F1 litters. Only F0 females received POPs or supplemented FA diet.

F0 females were housed two/cage for 10 days of acclimatization and then randomly assigned to four treatment groups (n = 6; Fig. 2): (1) Control group (“CTRL”) were fed the control diet (2 mg FA/kg diet described above); (2) POPs group (“POPs”) received the POPs mixture (Table 4) by gavage and fed the control diet; (3) FA-supplemented group (“FA”) were fed the FA-supplemented diet (6 mg FA/kg diet); (4) POPs and FA supplemented group (“POPsFA”) were gavaged with the POPs mixture and fed the FA-supplemented diet. All groups were weighed and gavaged thrice weekly with corn oil (CTRL and FA groups) or the POPs mixture (POPs and POPsFA groups) for five weeks before mating with untreated males (aged 90 days, Charles River). Mating was confirmed by the presence of sperm in vaginal smears and pregnant females were then housed individually. Gavage continued until the birth of F1 pups. After parturition, all F0 females received the control diet. Pups were weaned at Postnatal Day (PND) 21 and housed two/cage (n = 12). All subsequent generations received the control diet and no additional treatment.

At PND 90, randomly selected F1 males (two/F0 dam) were mated with untreated females (aged 63–74 days, Charles River) to generate F2 (Fig. 2). Likewise, F3 and F4 lineages were generated. After 36 h of pregnancy, females were sacrificed to collect two cell embryos (n = 12). At Gestational Day (GD) 19.5, females were sacrificed to collect fetuses (n = 6) and the remaining females gave birth (n = 6). Rats were anesthetized with 3% isoflurane then euthanized by exsanguination via cardiac puncture and CO_2_ asphyxiation. F4 adult males were not mated.

The body weights of the animals were closely monitored thrice weekly to ensure animal welfare. No apparent signs of general or systemic toxicity, such as behavioral changes or increased excitability, were observed. Weight gain increased over the nine weeks of treatment for all generations (Supplementary Fig. 1).

### Assessment of sperm concentration, morphology and viability

Adult males at PND 150 (n = 12 males) were euthanized to collect the *cauda* epididymides and sperm obtained by diffusion^66^ into M199 medium (GIBCO®, ThermoFisher Scientific, Mississauga, ON, Canada) at 37 °C for 30 min. Simultaneously, testes were collected, flash frozen and stored at −80 °C for spermatid counts.

*Cauda* epididymal sperm concentrations were counted using a hemocytometer (Bright-Line™, Sigma-Aldrich, Oakville, ON, Canada) by diluting 1/50 in fixation medium^67^. At least 200 spermatozoa/replicate were counted. Sperm counts were normalized by epididymal weight.

Sperm samples (50 µl) were further diluted in 450 µl warmed M199 medium. To assess morphology, sperm were smeared on a slide and dipped in SpermBlue fixative as previously reported^68^ (Ref. SB-250-N, ©MICROPTIC 2018, Spain). To evaluate viability, sperm were mixed with equal parts eosin-nigrosin stain^67^. Slides were prepared in duplicate. Morphology and viability were evaluated on 100 spermatozoa/slide using phase-contrast microscopy (400X).

Testicular spermatid counts were obtained using frozen testes (n = 4). The tunica albuginea was removed as described in Seung, Wolfe^69^. Weighted testes were homogenized in 10% DMSO/0.9% NaCl using a Polytron TissueMiser (Fisher Scientific, Pittsburgh, PA) and sonicated (Labsonic M, Sartorius Stedim, Oakville, ON, Canada) for 1 min at 40%. Spermatids were visualized using 0.1% trypan blue staining and counted by hemacytometry. Spermatid production was calculated as the mean count of both hemacytometer chambers divided by 0.4·10^−5^ ml X dilution volume (0.4·10^−5^ ml is the suspension volume used per chamber).

### Sperm motility analysis

Sperm motility (n = 12) was assessed by computer-assisted sperm analysis (CASA) with a CEROS analyzer (Version. 12 CEROS, Hamilton Thorne Research, Beverly, MA, USA). After incubation at 37 °C for 30 min, samples were loaded into 100 µm Rat Toxicology Slides (Leja Compagny, #SC 100-01-02-B, B.V., The Netherlands) and assessed for motility, progressive motility, average path velocity (VAP), linear velocity (VSL), curvilinear velocity (VCL), mean amplitude of lateral head displacement (ALH), frequency of head displacement (BCF), straightness coefficient (STR) and linearity coefficient (LIN). The following settings were used: frame rate, 60 Hz; frames acquired, 30; minimum contrast, 80 and minimum cell size, 7^70^. A minimum of 200 spermatozoa from five different fields was assessed.

### RNA analysis from two-cell embryos

Two-cell embryos were collected from multiple pregnancies sired by PND 90 males (n = 12) to examine the transcriptomes of F2 to F4 two-cell embryos. The untreated females were super-ovulated by intraperitoneal injection with 150 IU/kg PMSG (CDMV, Québec, Canada) followed 48 h later by 300 IU/kg hCG (CDMV, Québec, Canada). After hCG, super-ovulated females were combined with PND 90 F1, F2 or F3 males. Females were sacrificed 36 h after copulation and ovaries plus oviducts were collected in 37 °C M2 medium (Sigma, M7167). Using a pre-heated IVF Work Station (Origio Midatlantic Devices, NJ, USA), ovaries were separated from oviducts and small incisions were made to release the two-cell embryos. With a 100 µM Cook® Flexipet® Pipette (Cook Medical LLC, Bloomington, IN, USA), two-cell embryos were collected and stored at −80 °C until all embryos were obtained. To ensure sufficient material, 15 embryos from three sires (descended from different F0 dams) within each treatment were pooled for total RNA extraction. For each treatment, three replicates were assessed. From each pool, total RNA was extracted using the PicoPure™ RNA Isolation Kit (ThermoFisher Scientific, Mississauga, ON, Canada).

### Two-cell embryo RNA sequencing

cDNA libraries were constructed using SMART-Seq v4 Ultra low input RNA Kit (Clontech Laboratories, Takara Bio Company; CA, USA). First-strand cDNA was synthesized using total RNA from 15 two-cell embryos by the 3′ SMART-Seq CDS Primer II A and template switching was performed by SMART-Seq v4 Oligonucleotide at the 5′ end of the transcript. cDNA from SMART sequences was amplified by PCR Primer II A. After 14 cycles of long-distance PCR, amplified cDNA was purified using the Agencourt AMPure XP Kit (Beckman Coulter, Cat.No. A63882). The quality of cDNA construction was validated using Agilent Tapestation 2200 system. Final libraries for Illumina Next Generation sequencing were prepared with 150 pg of cDNA using Nextera XT DNA Library Preparation Kits (Illumina Inc., San Diego, CA, USA). Twenty-four libraries with unique indices were pooled in equimolar ratio and sequenced for paired-end 125 pb sequencing using three lanes of a high output flow cell on an Illumina HiSeq. 2500 V4 System. The average insert size for the paired-end libraries was 225 bp. Reads were trimmed using Trimmomatic v0.36 with the following options: TRAILING:30, SLIDINGWINDOW:4:20 and MINLEN:30. All other options used the default values. Quality checks were performed on raw and trimmed data using FastQC v0.11.5 and MultiQC v1.5. Quantification was performed with Kallisto v0.44 and differential expression analysis using R v3.5.0 using the DESeq. 2 v1.20.0. For subsequent analysis, only genes with a normalized count of >10, *p* value ≤ 0.05, Discovery Rate (FDR) ≤5% and −2≥ |Log2 Fold change| ≥2 (CTRL vs. other treatments) were included. Ingenuity® Pathway Analysis (IPA®, Ingenuity Systems Inc., Redwood City, CA) was used to identify gene ontology pathways altered by the significantly DE genes.

### Assessment of pregnancy outcomes (F1-F3 males)

Fertility parameters were assessed as a function of the father’s treatment lineage. Fetal analysis at GD 19.5, sex ratio and number of fetuses, *corpora lutea* and implantation sites were assessed to calculate the fertility rate (# fetuses/# *corpora lutea*)*100, pre-implantation losses ((# *corpora lutea* – # implantation sites)/# *corpora lutea*)*100 and post-implantation losses ((# implantation sites - # fetuses)/# implantation sites)*100. Litter size and number of dead and live pups were noted on PND 0, PND 6 and PND 11. Sex was confirmed by genital observation at PND 21.

### Statistical analysis

Data were analyzed using SAS University (Copyright © SAS Institute Inc., SAS Campus Drive, Cary, North Carolina 27513, USA) using *Mixed* Procedure with a one-way analysis of variance (ANOVA) in 2 × 2 factorial design. Main effects of POPs, FA and interaction (POPs*FA) were considered. The number of pups/litter and the F0 females were also included in the expanded model and excluded when not significant. To increase diversity and minimize the effect of the F0 dams, male lineages were derived from an equivalent number of the F0 founder females throughout the four generations. Differences were considered significant at *p* ≤ 0.05.

## Supplementary information

Dataset 1

## References

[1] Wohrnschimmel H (2016). Ten years after entry into force of the Stockholm Convention: What do air monitoring data tell about its effectiveness?. Environ Pollut.

[2] NCP, *Canadian Arctic Contaminants Assessment Report III - Persistent Organic Pollutants in Canada’s North*, in *Northern Contaminants Program*, K.-K.P. Muir D, Stow J., Editor. 2013, Gouvernment of Canada: Canada. p. 492.

[3] Zoeller RT (2012). Endocrine-disrupting chemicals and public health protection: a statement of principles from The Endocrine Society. Endocrinology.

[4] Chia Sin-Eng (2000). Endocrine disruptors and male reproductive function - a short review. International Journal of Andrology.

[5] Hauser R (2002). Environmental organochlorines and semen quality: results of a pilot study. Environ Health Perspect.

[6] Latif, T. *et al*. Semen quality is a predictor of subsequent morbidity. A Danish cohort study of 4,712 men with long-term follow-up. *Am J Epidemiol* (2017). 10.1093/aje/kwx067 28498890

[7] Eisenberg ML (2016). Increased risk of incident chronic medical conditions in infertile men: analysis of United States claims data. Fertil Steril.

[8] Jensen TK (2009). Good semen quality and life expectancy: a cohort study of 43,277 men. Am J Epidemiol.

[9] Consales C (2016). Exposure to persistent organic pollutants and sperm DNA methylation changes in Arctic and European populations. Environ Mol Mutagen.

[10] Vested A (2014). Persistent organic pollutants and male reproductive health. Asian J Androl.

[11] Skinner MK (2013). Environmentally induced transgenerational epigenetic reprogramming of primordial germ cells and the subsequent germ line. PLoS One.

[12] Veenendaal MV (2013). Transgenerational effects of prenatal exposure to the 1944-45 Dutch famine. Bjog.

[13] Guerrero-Bosagna C, Skinner MK (2014). Environmentally induced epigenetic transgenerational inheritance of male infertility. Curr Opin Genet Dev.

[14] Sharp GC, Lawlor DA, Richardson SS (2018). It’s the mother!: How assumptions about the causal primacy of maternal effects influence research on the developmental origins of health and disease. Soc Sci Med.

[15] Braun JM, Messerlian C, Hauser R (2017). Fathers Matter: Why It’s Time to Consider the Impact of Paternal Environmental Exposures on Children’s Health. Curr Epidemiol Rep.

[16] Soubry A (2015). Epigenetic inheritance and evolution: A paternal perspective on dietary influences. Prog Biophys Mol Biol.

[17] Czeizel AE, Dudas I (1992). Prevention of the first occurrence of neural-tube defects by periconceptional vitamin supplementation. N Engl J Med.

[18] MRC, Prevention of neural tube defects: results of the Medical Research Council Vitamin Study. MRC Vitamin Study Research Group. *Lancet*., **338**(8760), 131–7 (1991). 1677062

[19] Canada, G. o. Healthy Pregnancy. 2001 [cited 2011; Available from, https://www.canada.ca/en/health-canada/services/healthy-living/healthy-pregnancy.html.

[20] Canada, G. o. Regulatory impact analysis statement, C.G.P.I.-S. Instruments, Editor. 1998, Canadian Government: Canada. p. 3029–3033.

[21] Lambrot R (2013). Low paternal dietary folate alters the mouse sperm epigenome and is associated with negative pregnancy outcomes. Nat Commun.

[22] Swayne BG (2012). Investigating the effects of dietary folic acid on sperm count, DNA damage and mutation in Balb/c mice. Mutat Res.

[23] Wallock LM (2001). Low seminal plasma folate concentrations are associated with low sperm density and count in male smokers and nonsmokers. Fertil Steril.

[24] Shorter KR, Felder MR, Vrana PB (2015). Consequences of dietary methyl donor supplements: Is more always better?. Progress in Biophysics and Molecular Biology.

[25] Anas MK (2005). In utero and lactational exposure to an environmentally relevant organochlorine mixture disrupts reproductive development and function in male rats. Biol Reprod.

[26] Oswald J (2000). Active demethylation of the paternal genome in the mouse zygote. Curr Biol.

[27] Santos F (2002). Dynamic reprogramming of DNA methylation in the early mouse embryo. Dev Biol.

[28] Mumford SL (2015). Persistent organic pollutants and semen quality: The LIFE Study. Chemosphere.

[29] Swayne BG (2012). Supplemental dietary folic acid has no effect on chromosome damage in erythrocyte progenitor cells of mice. J Nutr.

[30] Bonde JP (2016). The epidemiologic evidence linking prenatal and postnatal exposure to endocrine disrupting chemicals with male reproductive disorders: a systematic review and meta-analysis. Hum Reprod Update.

[31] Spano M (2005). Exposure to PCB and p, p′-DDE in European and Inuit populations: impact on human sperm chromatin integrity. Hum Reprod.

[32] Toft G (2014). Persistent organochlorine pollutants and human reproductive health. Dan Med J.

[33] Aneck-Hahn NH (2007). Impaired semen quality associated with environmental DDT exposure in young men living in a malaria area in the Limpopo Province, South Africa. J Androl.

[34] De Jager C (2006). Reduced seminal parameters associated with environmental DDT exposure and p,p’-DDE concentrations in men in Chiapas, Mexico: a cross-sectional study. J Androl.

[35] Skakkebaek NE (2016). Male Reproductive Disorders and Fertility Trends: Influences of Environment and Genetic Susceptibility. Physiol Rev.

[36] Hass U (2012). Adverse effects on sexual development in rat offspring after low dose exposure to a mixture of endocrine disrupting pesticides. Reprod Toxicol.

[37] Sanabria M (2016). Sperm quality and fertility in rats after prenatal exposure to low doses of TCDD: A three-generation study. Reprod Toxicol.

[38] Vidaeff AC, Sever LE (2005). In utero exposure to environmental estrogens and male reproductive health: a systematic review of biological and epidemiologic evidence. Reprod Toxicol.

[39] Vested A (2013). Associations of in utero exposure to perfluorinated alkyl acids with human semen quality and reproductive hormones in adult men. Environ Health Perspect.

[40] Vieira ML (2017). Chronic exposure to the fungicide propiconazole: Behavioral and reproductive evaluation of F1 and F2 generations of male rats. Toxicology.

[41] Ly L, Chan D, Trasler JM (2015). Developmental windows of susceptibility for epigenetic inheritance through the male germline. Semin Cell Dev Biol.

[42] Alegria-Torres JA, Baccarelli A, Bollati V (2011). Epigenetics and lifestyle. Epigenomics.

[43] Grova N (2019). Epigenetic and Neurological Impairments Associated with Early Life Exposure to Persistent Organic Pollutants. Int J Genomics.

[44] Song Y (2014). Transgenerational impaired male fertility with an Igf2 epigenetic defect in the rat are induced by the endocrine disruptor p,p’-DDE. Hum Reprod.

[45] De Wals P (2007). Reduction in neural-tube defects after folic acid fortification in Canada. N Engl J Med.

[46] Wilson RD (2015). Pre-conception Folic Acid and Multivitamin Supplementation for the Primary and Secondary Prevention of Neural Tube Defects and Other Folic Acid-Sensitive Congenital Anomalies. J Obstet Gynaecol Can.

[47] Salarkia E (2017). Effects of administration of co-trimoxazole and folic acid on sperm quality and histological changes of testes in male rats. Int J Reprod Biomed (Yazd).

[48] Dolinoy DC, Huang D, Jirtle RL (2007). Maternal nutrient supplementation counteracts bisphenol A-induced DNA hypomethylation in early development. Proc Natl Acad Sci USA.

[49] Gomaa AM, Abou Khalil NS, Abdel-Ghani MA (2017). The protective role of folic acid against testicular dysfunction in lead-intoxicated rat model. Gen Physiol Biophys.

[50] Sakr S (2018). Beneficial effects of folic acid on the kidneys and testes of adult albino rats after exposure to methomyl. Toxicol Res (Camb).

[51] Gules O (2019). Effects of folic acid on testicular toxicity induced by bisphenol-A in male Wistar rats. Biotech Histochem.

[52] Deng Y (2017). High Serum Folate Is Associated with Brain Atrophy in Older Diabetic People with Vitamin B12 Deficiency. J Nutr Health Aging.

[53] Aarabi M (2015). High-dose folic acid supplementation alters the human sperm methylome and is influenced by the MTHFR C677T polymorphism. Hum Mol Genet.

[54] Schaible TD (2011). Maternal methyl-donor supplementation induces prolonged murine offspring colitis susceptibility in association with mucosal epigenetic and microbiomic changes. Hum Mol Genet.

[55] Barua S (2016). DNA Methylation Profiling at Single-Base Resolution Reveals Gestational Folic Acid Supplementation Influences the Epigenome of Mouse Offspring Cerebellum. Front Neurosci.

[56] Toft G (2004). Epidemiological evidence on reproductive effects of persistent organochlorines in humans. Reprod Toxicol.

[57] Savitz DA (2014). Persistent organochlorines and hypertensive disorders of pregnancy. Environ Res.

[58] Heard E, Martienssen RA (2014). Transgenerational epigenetic inheritance: myths and mechanisms. Cell.

[59] Latif T (2018). Semen quality associated with subsequent hospitalizations - Can the effect be explained by socio-economic status and lifestyle factors?. Andrology.

[60] Levine H (2017). Temporal trends in sperm count: a systematic review and meta-regression analysis. Hum Reprod Update.

[61] Chan YM, MacFarlane AJ, O’Connor DL (2015). Modeling Demonstrates That Folic Acid Fortification of Whole-Wheat Flour Could Reduce the Prevalence of Folate Inadequacy in Canadian Whole-Wheat Consumers. J Nutr.

[62] MacFarlane AJ, Greene-Finestone LS, Shi Y (2011). Vitamin B-12 and homocysteine status in a folate-replete population: results from the Canadian Health Measures Survey. Am J Clin Nutr.

[63] Maurice C (2018). Prenatal exposure to an environmentally relevant mixture of Canadian Arctic contaminants decreases male reproductive function in an aging rat model. J Dev Orig Health Dis.

[64] Muir D (1999). Spatial and temporal trends and effects of contaminants in the Canadian Arctic marine ecosystem: a review. Sci Total Environ.

[65] Reeves PG, Nielsen FH, Fahey GC (1993). AIN-93 purified diets for laboratory rodents: final report of the American Institute of Nutrition ad hoc writing committee on the reformulation of the AIN-76A rodent diet. J Nutr.

[66] Klinefelter GR, Gray LE, Suarez JD (1991). The method of sperm collection significantly influences sperm motion parameters following ethane dimethanesulphonate administration in the rat. Reprod Toxicol.

[67] WHO, WHO Laboratory Manual for the Examination and Processing of Human Semen. 2010, World Health Organisation: Genève. p. 286.

[68] van der Horst G, Maree L (2009). SpermBlue: a new universal stain for human and animal sperm which is also amenable to automated sperm morphology analysis. Biotech Histochem.

[69] Seung H, Wolfe G, Rocca M (2003). Performing a Testicular Spermatid Head Count. Current Protocols in Toxicology.

[70] Zhou Y (2008). An epididymis-specific secretory protein HongrES1 critically regulates sperm capacitation and male fertility. PLoS One.

