# Supplementary material for: Prenatal Exposure to Environmentally-Relevant Contaminants Perturbs Male Reproductive Parameters Across Multiple Generations that are Partially Protected by Folic Acid Supplementation
Source: Sci Rep. 2019 Sep 25;9:13829. doi: 10.1038/s41598-019-50060-z (PMC6761122; doi:10.1038/s41598-019-50060-z)

Prenatal Exposure to Environmentally-Relevant Contaminants Perturbs Male Reproductive Parameters Across Multiple Generations that are Partially Protected by Folic Acid Supplementation.

Maryse Lessard[1*], Pauline M. Herst[1*], Phanie L. Charest[1], Pauline Navarro[2], Charles Joly-Beauparlant[3], Arnaud Droit[3], Sarah Kimmins[4], Jacquetta Trasler[5], Marie-Odile Benoit-Biancamano[6], Amanda J. MacFarlane[7], Mathieu Dalvai[1] and Janice L. Bailey[1]

Supplementary Table 1. Fold change of all **s**hared significantly differentially expressed (DE) genes between POPs, FA and POPsFA in F2 (29 genes total), F3 (2 genes total) and F4 (288 genes total).

F2

Ensembl ID	Official gene symbol	POPs	FA	POPsFA
ENSRNOG00000003160	RragB	7.48	8.01	7.01
ENSRNOG00000017100	LOC108348078	9.81	6.46	7.21
ENSRNOG00000026235	Hk3	-6.05	4.51	3.48
ENSRNOG00000017208	Cspg4	-5.91	5.69	4.69
ENSRNOG00000010253	Cd163	-5.76	4.22	3.47
ENSRNOG00000021424	Cd300lf	-5.00	2.63	2.29
ENSRNOG00000033787	Adamtsl5	-4.80	3.25	2.53
ENSRNOG00000027811	Lilrb4	-3.85	4.79	3.02
ENSRNOG00000010183	Fam198b	-3.58	3.65	2.91
ENSRNOG00000021161	Fermt3	-3.42	3.97	2.84
ENSRNOG00000016294	Cd4	-3.32	3.13	2.27
ENSRNOG00000053260	Lilrb3a	-3.04	2.90	2.34
ENSRNOG00000050430	Vav1	-2.87	3.38	2.30
ENSRNOG00000016512	Sema3b	-2.77	2.14	2.23
ENSRNOG00000046663	LOC100911825	-2.64	4.37	3.05
ENSRNOG00000039390	Slc37a2	-2.63	3.17	2.08
ENSRNOG00000012749	C1qb	-2.56	3.55	2.77
ENSRNOG00000012807	C1qa	-2.55	3.51	2.55
ENSRNOG00000028668	Slc28a2	-2.47	3.54	2.78
ENSRNOG00000003622	Cybb	-2.45	3.76	2.73
ENSRNOG00000006094	Cd44	-2.44	3.78	2.87
ENSRNOG00000033564	Cfd	-2.42	4.53	3.53
ENSRNOG00000025001	Pcolce	-2.39	2.28	2.79
ENSRNOG00000005825	Lyz2	-2.34	3.52	2.64
ENSRNOG00000009848	Il18	-2.26	2.62	2.02

ENSRNOG00000009822	Tlr2	-2.19	2.59	2.02
ENSRNOG00000054251	Clec7a	-2.12	3.48	2.60
ENSRNOG00000052219	Gm2a	-2.07	2.98	2.72
ENSRNOG00000019890	Folr2	-2.05	5.07	4.07

F3

**Ensembl ID**	**Official gene symbol**	**POPs**	**FA**	**POPsFA**
ENSRNOG00000006368	Lrrn3	-4.12	-3.16	-3.39
ENSRNOG00000030183	Plod2	-3.64	-3.87	-3.23

F4

Ensembl ID	Official gene symbol	POPs	FA	POPsFA
ENSRNOG00000047388	Vom2r4	-11.21	-10.39	-11.00
ENSRNOG00000050370	Vom2r6	-9.22	-10.22	-10.27
ENSRNOG00000012057	Olig3	-9.40	-9.75	-8.73
ENSRNOG00000016421	Tyr	-10.53	-9.72	-10.55
ENSRNOG00000060573	AABR07004437.1	-7.29	-9.28	-7.48
ENSRNOG00000002167	Dmp1	-8.28	-9.25	-10.31
ENSRNOG00000032626	Mmp3	-7.89	-9.18	-7.76
ENSRNOG00000051929	Phox2b	-6.92	-9.17	-9.54
ENSRNOG00000054957	Sfrp4	-9.54	-8.85	-13.34
ENSRNOG00000019377	Amh	-7.75	-8.84	-8.44
ENSRNOG00000021108	Slc22a12	-9.13	-8.79	-9.11
ENSRNOG00000058285	Nme8	-8.47	-8.44	-8.62
ENSRNOG00000012671	Gan	-8.03	-8.23	-9.21
ENSRNOG00000048390	AABR07006536.1	-8.25	-7.86	-8.31
ENSRNOG00000058560	Col2a1	-7.66	-7.82	-7.47
ENSRNOG00000054716	AABR07015907.1	-7.44	-7.70	-8.40
ENSRNOG00000051678	AABR07012475.1	-8.95	-7.70	-7.93
ENSRNOG00000053805	Akain1	-8.18	-7.70	-8.50
ENSRNOG00000011892	Slc36a2	-7.78	-7.49	-6.17
ENSRNOG00000047891	Foxg1	-6.62	-7.40	-7.28
ENSRNOG00000024904	Pla2g4e	-6.79	-7.34	-9.46
ENSRNOG00000032745	Slc17a3	-6.62	-7.22	-7.61
ENSRNOG00000049019	Tmem170a	-7.04	-7.19	-7.08
ENSRNOG00000016362	Gpr4	-8.13	-7.14	-8.13
ENSRNOG00000057392	AABR07042859.1	-9.31	-7.06	-9.80
ENSRNOG00000022044	Cabp4	-7.25	-6.92	-6.03
ENSRNOG00000046611	Dzip1-ps1	-7.97	-6.87	-6.77
ENSRNOG00000045689	AABR07037645.1	-8.15	-6.87	-7.28
ENSRNOG00000057463	AABR07008293.4	-7.96	-6.85	-7.95
ENSRNOG00000000196	Cyp19a1	-7.21	-6.82	-7.57
ENSRNOG00000006741	Podnl1	-7.37	-6.79	-7.27
ENSRNOG00000029876	Gucy1a2	-7.33	-6.74	-6.39
ENSRNOG00000042897	Nmrk2	-6.84	-6.63	-7.06
ENSRNOG00000042222	RGD1563562	-7.68	-6.63	-8.01
ENSRNOG00000012428	Maf	-6.41	-6.61	-7.18
ENSRNOG00000033883	Stard8	-6.62	-6.58	-6.43
ENSRNOG00000038068	Pcdh9	-8.36	-6.58	-7.43
ENSRNOG00000006776	Smyd1	-7.31	-6.54	-7.59

ENSRNOG00000059091	Potem	-8.65	-6.51	-6.77
ENSRNOG00000052093	Smok2a	-7.48	-6.38	-6.96
ENSRNOG00000049504	AABR07009221.1	-7.04	-6.35	-7.32
ENSRNOG00000017333	Syt4	-9.97	-6.35	-6.06
ENSRNOG00000020097	Inha	-6.73	-6.33	-6.65
ENSRNOG00000018286	Chrna1	-6.24	-6.30	-6.78
ENSRNOG00000008121	Slc13a1	-5.73	-6.28	-5.58
ENSRNOG00000015002	Abhd15	-5.69	-6.26	-6.98
ENSRNOG00000045797	Lep	-6.50	-6.24	-6.25
ENSRNOG00000051598	AABR07013583.1	-7.11	-6.19	-7.28
ENSRNOG00000004679	Fign	-7.41	-6.17	-4.89
ENSRNOG00000032706	Kcnk18	-6.61	-6.16	-6.19
ENSRNOG00000055570	AABR07034940.2	-5.86	-6.15	-6.23
ENSRNOG00000030156	LOC102547344	-8.42	-6.12	-7.41
ENSRNOG00000049016	AABR07048012.1	-7.43	-6.11	-7.02
ENSRNOG00000049451	AABR07048013.1	-7.43	-6.11	-7.02
ENSRNOG00000010348	Cacna1f	-6.64	-6.10	-5.26
ENSRNOG00000046711	AC128353.1	-5.89	-6.05	-6.99
ENSRNOG00000057039	AC124874.1	-7.41	-6.04	-7.39
ENSRNOG00000003997	Pld5	-5.72	-6.02	-5.32
ENSRNOG00000037435	Tmem196	-5.28	-6.02	-5.49
ENSRNOG00000032558	AABR07030263.1	-5.89	-6.01	-6.96
ENSRNOG00000060832	AC240408.2	-7.20	-5.98	-7.23
ENSRNOG00000032002	Hapln1	-6.22	-5.97	-6.58
ENSRNOG00000042535	AABR07010907.1	-7.34	-5.97	-6.66
ENSRNOG00000054519	AABR07053179.1	-7.32	-5.97	-7.27
ENSRNOG00000014253	Pax2	-6.58	-5.95	-6.30
ENSRNOG00000019193	Stx1b	-6.38	-5.94	-6.39
ENSRNOG00000050847	AABR07027902.1	-7.00	-5.94	-6.99
ENSRNOG00000051648	AABR07016566.1	-5.98	-5.90	-6.39
ENSRNOG00000059400	AABR07070796.1	-7.53	-5.85	-7.20
ENSRNOG00000058491	AABR07017104.1	-7.09	-5.83	-7.07
ENSRNOG00000057038	AABR07029803.1	-7.78	-5.82	-6.94
ENSRNOG00000016679	Tmc7	-6.71	-5.74	-6.11
ENSRNOG00000017686	Pi15	-7.39	-5.73	-6.56
ENSRNOG00000014871	Zic4	-5.29	-5.73	-5.95
ENSRNOG00000008431	Gabbr2	-6.36	-5.73	-6.38
ENSRNOG00000010106	Faxc	-6.31	-5.70	-6.39
ENSRNOG00000017197	Pdgfb	-5.74	-5.69	-4.91
ENSRNOG00000003899	Krt14	-7.03	-5.69	-5.42
ENSRNOG00000045560	Gvin1	-4.90	-5.67	-6.26
ENSRNOG00000007942	Fscn3	-6.26	-5.67	-5.70
ENSRNOG00000031879	AABR07026048.1	-5.86	-5.67	-5.27
ENSRNOG00000000871	Cd40lg	-5.83	-5.67	-5.88

ENSRNOG00000052460	AABR07004812.2	-6.58	-5.63	-6.82
ENSRNOG00000058101	AABR07067387.1	-6.72	-5.62	-6.73
ENSRNOG00000024789	LOC499219	-6.59	-5.48	-6.49
ENSRNOG00000008053	Atp8a2	-5.35	-5.46	-5.18
ENSRNOG00000053122	Scn1a	-5.74	-5.43	-6.83
ENSRNOG00000053229	AABR07007905.2	-6.64	-5.38	-6.41
ENSRNOG00000013312	Kcnt2	-4.65	-5.38	-4.57
ENSRNOG00000005749	Foxred2	-5.85	-5.37	-6.10
ENSRNOG00000010834	Mpped1	-5.73	-5.32	-6.30
ENSRNOG00000030266	Plekhg2	-4.98	-5.28	-6.12
ENSRNOG00000004623	Rag2	-5.81	-5.27	-6.51
ENSRNOG00000002624	Edaradd	-6.06	-5.24	-5.14
ENSRNOG00000010478	LOC299282	-6.47	-5.23	-6.18
ENSRNOG00000026302	Lrrn4cl	-5.86	-5.22	-5.99
ENSRNOG00000047671	AABR07060588.2	-5.79	-5.21	-6.22
ENSRNOG00000042860	Pappa2	-6.91	-5.20	-4.73
ENSRNOG00000038369	AABR07038477.1	-5.02	-5.19	-5.65
ENSRNOG00000002265	Casr	-6.21	-5.19	-6.06
ENSRNOG00000055846	AABR07013065.1	-6.23	-5.12	-6.11
ENSRNOG00000060553	AC240408.1	-5.67	-5.11	-6.33
ENSRNOG00000012165	Ccdc129	-5.45	-5.10	-6.52
ENSRNOG00000008644	Nkx2-1	-6.20	-5.09	-6.17
ENSRNOG00000057092	Slfn4	-4.61	-5.06	-6.71
ENSRNOG00000049140	AABR07060678.1	-6.14	-5.06	-6.13
ENSRNOG00000056033	AC111632.2	-6.02	-5.03	-6.10
ENSRNOG00000011258	Masp2	-4.80	-5.03	-4.91
ENSRNOG00000058277	AC080157.1	-5.55	-4.98	-5.53
ENSRNOG00000028112	LOC499796	-6.01	-4.97	-6.84
ENSRNOG00000060618	AABR07027267.1	-5.72	-4.95	-5.89
ENSRNOG00000059691	AABR07033318.1	-5.77	-4.95	-5.73
ENSRNOG00000002723	Sele	-5.58	-4.93	-6.53
ENSRNOG00000010798	Blk	-5.42	-4.93	-5.79
ENSRNOG00000016760	Kctd19	-4.81	-4.90	-4.69
ENSRNOG00000047765	AABR07010682.1	-5.93	-4.89	-5.95
ENSRNOG00000036661	Rab40b	-5.54	-4.89	-4.43
ENSRNOG00000012920	Col9a1	-5.36	-4.88	-5.27
ENSRNOG00000061900	AABR07047771.1	-5.62	-4.88	-5.86
ENSRNOG00000011177	Sftpc	-5.54	-4.86	-5.49
ENSRNOG00000051345	AABR07048469.2	-5.20	-4.86	-4.91
ENSRNOG00000040314	AABR07034637.1	-5.88	-4.85	-5.88
ENSRNOG00000008050	Stac3	-4.16	-4.85	-5.25
ENSRNOG00000008367	Krt86	-5.60	-4.84	-5.90
ENSRNOG00000034226	LOC100362981	-6.80	-4.84	-6.42
ENSRNOG00000057744	AC112355.1	-5.37	-4.83	-5.60

ENSRNOG00000000618	Mdga2	-5.52	-4.83	-4.63
ENSRNOG00000022565	Lrrc25	-5.45	-4.80	-5.33
ENSRNOG00000053741	AABR07052664.1	-5.73	-4.78	-5.57
ENSRNOG00000010128	Slc27a2	-4.35	-4.76	-4.70
ENSRNOG00000061132	LOC103694210	-5.44	-4.75	-5.74
ENSRNOG00000011068	Papss2	-5.58	-4.75	-5.78
ENSRNOG00000050885	Sult1c2	-4.73	-4.72	-3.18
ENSRNOG00000032995	AABR07025089.1	-5.83	-4.68	-5.51
ENSRNOG00000052774	AC123253.2	-5.38	-4.65	-5.60
ENSRNOG00000031202	Trim30c	-5.58	-4.62	-5.67
ENSRNOG00000029588	LOC100364769	-5.51	-4.62	-5.62
ENSRNOG00000017890	Crhbp	-4.65	-4.61	-5.24
ENSRNOG00000030538	Slco1b2	-5.21	-4.61	-5.54
ENSRNOG00000055015	AABR07015559.1	-5.86	-4.59	-5.31
ENSRNOG00000043357	Zfp407	-4.47	-4.58	-4.91
ENSRNOG00000024390	Osm	-5.84	-4.58	-6.30
ENSRNOG00000024569	Gimap9	-4.99	-4.57	-5.17
ENSRNOG00000030689	Ms4a6e	-5.28	-4.57	-5.55
ENSRNOG00000046547	Rbm24	-3.73	-4.55	-3.87
ENSRNOG00000049791	AABR07059552.1	-5.99	-4.55	-5.62
ENSRNOG00000025235	Tmem130	-5.09	-4.54	-4.31
ENSRNOG00000028908	Eppin	-4.38	-4.54	-4.62
ENSRNOG00000059027	AC098008.5	-5.73	-4.53	-5.37
ENSRNOG00000026238	RGD1562618	-4.43	-4.51	-4.66
ENSRNOG00000007307	Syde1	-4.86	-4.51	-5.54
ENSRNOG00000048305	AABR07013922.1	-6.16	-4.50	-5.44
ENSRNOG00000058003	Spon1	-5.00	-4.50	-5.51
ENSRNOG00000000378	AABR07044914.1	-5.38	-4.50	-5.50
ENSRNOG00000062191	AABR07036016.1	-4.98	-4.49	-4.45
ENSRNOG00000008587	Tek	-6.50	-4.49	-6.12
ENSRNOG00000004459	Sdr9c7	-5.24	-4.48	-5.21
ENSRNOG00000047234	AABR07057530.1	-4.87	-4.48	-4.73
ENSRNOG00000048917	Hoxa6	-3.84	-4.46	-4.24
ENSRNOG00000058209	AABR07063893.1	-5.80	-4.46	-5.61
ENSRNOG00000059506	AC124926.2	-5.67	-4.45	-5.55
ENSRNOG00000052264	AABR07034393.1	-6.17	-4.44	-5.02
ENSRNOG00000059362	Has3	-4.66	-4.43	-4.35
ENSRNOG00000010997	Ednrb	-4.79	-4.43	-5.29
ENSRNOG00000058984	AC115181.1	-5.29	-4.42	-5.15
ENSRNOG00000046774	AABR07045307.1	-5.55	-4.41	-5.73
ENSRNOG00000061874	AABR07047036.1	-5.32	-4.39	-5.31
ENSRNOG00000013373	Rmt1	-4.81	-4.38	-4.35
ENSRNOG00000015691	Fam212b	-4.64	-4.38	-4.64
ENSRNOG00000015538	Abcd2	-5.29	-4.33	-5.08

ENSRNOG00000061625	AABR07048698.1	-5.46	-4.33	-5.34
ENSRNOG00000053337	Ly49s6	-5.48	-4.31	-5.47
ENSRNOG00000028072	Chit1	-4.88	-4.30	-5.25
ENSRNOG00000042471	RGD1560324	-5.63	-4.28	-5.34
ENSRNOG00000017833	Actn2	-3.86	-4.27	-4.72
ENSRNOG00000007059	Atp1b4	-4.74	-4.24	-4.90
ENSRNOG00000007664	Tnfrsf13c	-4.33	-4.21	-4.64
ENSRNOG00000060489	AABR07015639.1	-5.16	-4.21	-5.28
ENSRNOG00000001189	Sik1	-4.07	-4.20	-4.72
ENSRNOG00000037684	RGD1566159	-4.27	-4.19	-4.66
ENSRNOG00000039593	Ecscr	-4.02	-4.18	-4.00
ENSRNOG00000050966	AABR07024757.1	-5.38	-4.18	-5.22
ENSRNOG00000059468	AABR07034573.3	-4.87	-4.17	-4.96
ENSRNOG00000008074	Cyp11a1	-3.94	-4.17	-3.63
ENSRNOG00000054385	Rhebl1	-3.92	-4.16	-3.71
ENSRNOG00000049314	AABR07069524.1	-4.91	-4.15	-4.97
ENSRNOG00000059786	AC107531.3	-4.39	-4.12	-4.48
ENSRNOG00000056490	AABR07013566.1	-5.31	-4.11	-4.99
ENSRNOG00000010853	Chrna7	-4.49	-4.11	-4.45
ENSRNOG00000061264	AABR07058091.2	-4.35	-4.09	-4.69
ENSRNOG00000008842	Cyp4a8	-4.63	-4.07	-4.69
ENSRNOG00000024482	Tnrc18	-4.36	-4.04	-4.08
ENSRNOG00000009043	Piwil4	-4.10	-4.04	-4.06
ENSRNOG00000022022	AC128967.1	-4.71	-4.04	-4.71
ENSRNOG00000053367	AABR07064257.1	-4.71	-4.03	-4.94
ENSRNOG00000047635	Tmem178b	-4.01	-4.03	-5.38
ENSRNOG00000060246	Klrd1	-4.77	-4.02	-4.67
ENSRNOG00000003669	Myocd	-4.64	-3.99	-4.82
ENSRNOG00000037452	AABR07042542.1	-5.11	-3.99	-5.02
ENSRNOG00000046505	Bend3	-3.72	-3.99	-4.19
ENSRNOG00000043188	AABR07000261.1	-4.12	-3.99	-4.50
ENSRNOG00000019057	Prkcq	-4.22	-3.97	-5.66
ENSRNOG00000037449	Pole	-3.67	-3.97	-3.92
ENSRNOG00000037997	Tssk2	-4.32	-3.97	-4.33
ENSRNOG00000058653	Tmem52b	-3.89	-3.97	-4.35
ENSRNOG00000050509	Ebi3	-3.36	-3.95	-4.01
ENSRNOG00000060946	LOC100910506	-4.39	-3.89	-4.09
ENSRNOG00000032361	AABR07038798.1	-4.47	-3.88	-4.48
ENSRNOG00000009832	Slc39a14	-3.74	-3.88	-3.80
ENSRNOG00000056187	AC132752.1	-4.44	-3.88	-4.47
ENSRNOG00000021663	RGD1561849	-4.01	-3.87	-3.56
ENSRNOG00000000010	Cbln1	-3.95	-3.85	-4.47
ENSRNOG00000059705	Elmo1	-4.09	-3.84	-4.06
ENSRNOG00000006509	Srgap3	-4.22	-3.81	-4.69

ENSRNOG00000038835	Cd86	-4.29	-3.80	-4.31
ENSRNOG00000029244	Pcdhb10	-5.13	-3.79	-4.65
ENSRNOG00000037275	Tlcd2	-4.12	-3.79	-4.58
ENSRNOG00000051802	AABR07010609.1	-4.83	-3.77	-4.88
ENSRNOG00000006783	Neb	-4.90	-3.73	-4.15
ENSRNOG00000026582	RGD1310212	-3.64	-3.71	-3.99
ENSRNOG00000051664	AABR07010878.1	-4.54	-3.70	-4.63
ENSRNOG00000058766	AABR07019341.2	-4.22	-3.69	-4.29
ENSRNOG00000061317	AABR07011733.1	-4.73	-3.69	-4.83
ENSRNOG00000052304	AC096201.1	-4.66	-3.67	-4.92
ENSRNOG00000046332	AABR07004812.1	-4.82	-3.62	-4.55
ENSRNOG00000028548	Ccl9	-4.18	-3.60	-4.66
ENSRNOG00000060998	AABR07029467.2	-4.31	-3.60	-4.34
ENSRNOG00000010975	Adnp	-3.38	-3.58	-3.42
ENSRNOG00000059588	AC113785.2	-4.33	-3.55	-4.32
ENSRNOG00000014137	Fbln1	-3.20	-3.54	-4.02
ENSRNOG00000015133	Kmt2a	-3.88	-3.48	-4.03
ENSRNOG00000031951	LOC100361645	-4.38	-3.48	-4.41
ENSRNOG00000016848	Fzd4	-3.97	-3.48	-3.99
ENSRNOG00000031579	LOC100363469	-4.22	-3.47	-4.43
ENSRNOG00000025648	Dhrs7l1	-4.13	-3.43	-4.40
ENSRNOG00000051932	AABR07016635.1	-4.89	-3.41	-4.46
ENSRNOG00000010889	Fbxw7	-3.73	-3.39	-3.09
ENSRNOG00000009826	Bche	-3.59	-3.29	-3.62
ENSRNOG00000000113	Elac1	-3.27	-3.28	-3.45
ENSRNOG00000034134	Cpm	-3.23	-3.27	-3.55
ENSRNOG00000013004	Akr1d1	-4.61	-3.27	-3.60
ENSRNOG00000059145	AABR07026137.2	-4.24	-3.26	-4.33
ENSRNOG00000001271	Card6	-3.50	-3.24	-3.76
ENSRNOG00000059322	N4bp2l1	-3.51	-3.23	-3.84
ENSRNOG00000036726	Cd300e	-4.11	-3.22	-3.54
ENSRNOG00000004697	Baalc	-3.49	-3.22	-3.72
ENSRNOG00000019590	Smg5	-3.77	-3.22	-4.09
ENSRNOG00000046353	LOC501406	-3.07	-3.20	-2.81
ENSRNOG00000005871	Il1rn	-3.44	-3.19	-3.09
ENSRNOG00000027468	Slc6a15	-3.25	-3.19	-3.90
ENSRNOG00000042741	Adgb	-3.55	-3.19	-3.32
ENSRNOG00000036604	Ifit2	-2.76	-3.17	-3.13
ENSRNOG00000020444	Hcn3	-3.60	-3.16	-3.96
ENSRNOG00000048904	AABR07004232.1	-3.82	-3.15	-3.92
ENSRNOG00000018839	Ntrk2	-3.41	-3.12	-3.45
ENSRNOG00000059252	AABR07057108.1	-4.13	-3.08	-3.95
ENSRNOG00000052847	AABR07025673.1	-3.70	-3.04	-4.03
ENSRNOG00000014452	Zfhx3	-2.70	-3.02	-3.12

ENSRNOG00000006649	Thrb	-3.58	-3.00	-3.11
ENSRNOG00000011991	Slc10a7	-2.76	-2.98	-2.99
ENSRNOG00000060742	AABR07029809.1	-4.03	-2.97	-4.00
ENSRNOG00000007548	Polr3f	-3.09	-2.95	-3.16
ENSRNOG00000050205	Afmid	-3.28	-2.94	-3.54
ENSRNOG00000030870	LOC102550396	-3.93	-2.94	-3.78
ENSRNOG00000048762	AABR07000534.1	-4.20	-2.90	-3.90
ENSRNOG00000051390	AABR07035064.1	-3.63	-2.89	-3.48
ENSRNOG00000057986	AABR07034980.2	-3.63	-2.89	-3.48
ENSRNOG00000046647	Impad1	-2.78	-2.86	-3.06
ENSRNOG00000000456	Psmb8	-3.21	-2.84	-3.05
ENSRNOG00000012439	Bid	-3.05	-2.81	-3.36
ENSRNOG00000036842	Smug1	-2.84	-2.68	-2.82
ENSRNOG00000005904	Cdc27	-2.48	-2.67	-2.84
ENSRNOG00000058408	AC108588.1	-3.75	-2.66	-3.52
ENSRNOG00000015517	Zfp444	-2.96	-2.66	-2.66
ENSRNOG00000017066	Zfp384	-2.62	-2.65	-2.63
ENSRNOG00000014288	Fn1	-2.70	-2.65	-3.06
ENSRNOG00000002866	Rassf6	-2.90	-2.63	-2.51
ENSRNOG00000011829	Rpgrip1l	-2.55	-2.59	-2.53
ENSRNOG00000037251	Zfp248	-2.55	-2.57	-2.69
ENSRNOG00000024120	Rxfp1	-2.79	-2.55	-3.09
ENSRNOG00000001548	Nfe2l2	-2.37	-2.50	-2.52
ENSRNOG00000013521	Dhfr	-2.41	-2.47	-2.74
ENSRNOG00000003132	Mip	-2.36	-2.40	-2.97
ENSRNOG00000008908	Slc35a1	-2.70	-2.32	-2.93
ENSRNOG00000027434	Fitm2	-2.46	-2.29	-2.49
ENSRNOG00000013624	Uevld	-2.70	-2.28	-2.83
ENSRNOG00000017672	Akr1c14	-2.49	-1.93	-2.51
ENSRNOG00000003160	RragB	4.67	5.03	4.59

Supplementary Figure 1. Weight monitoring of F0 female rats and F1 - F4 male offspring. The weight of the animals was closely monitored trice a week to ensure animal welfare. Mean weight is shown over time.

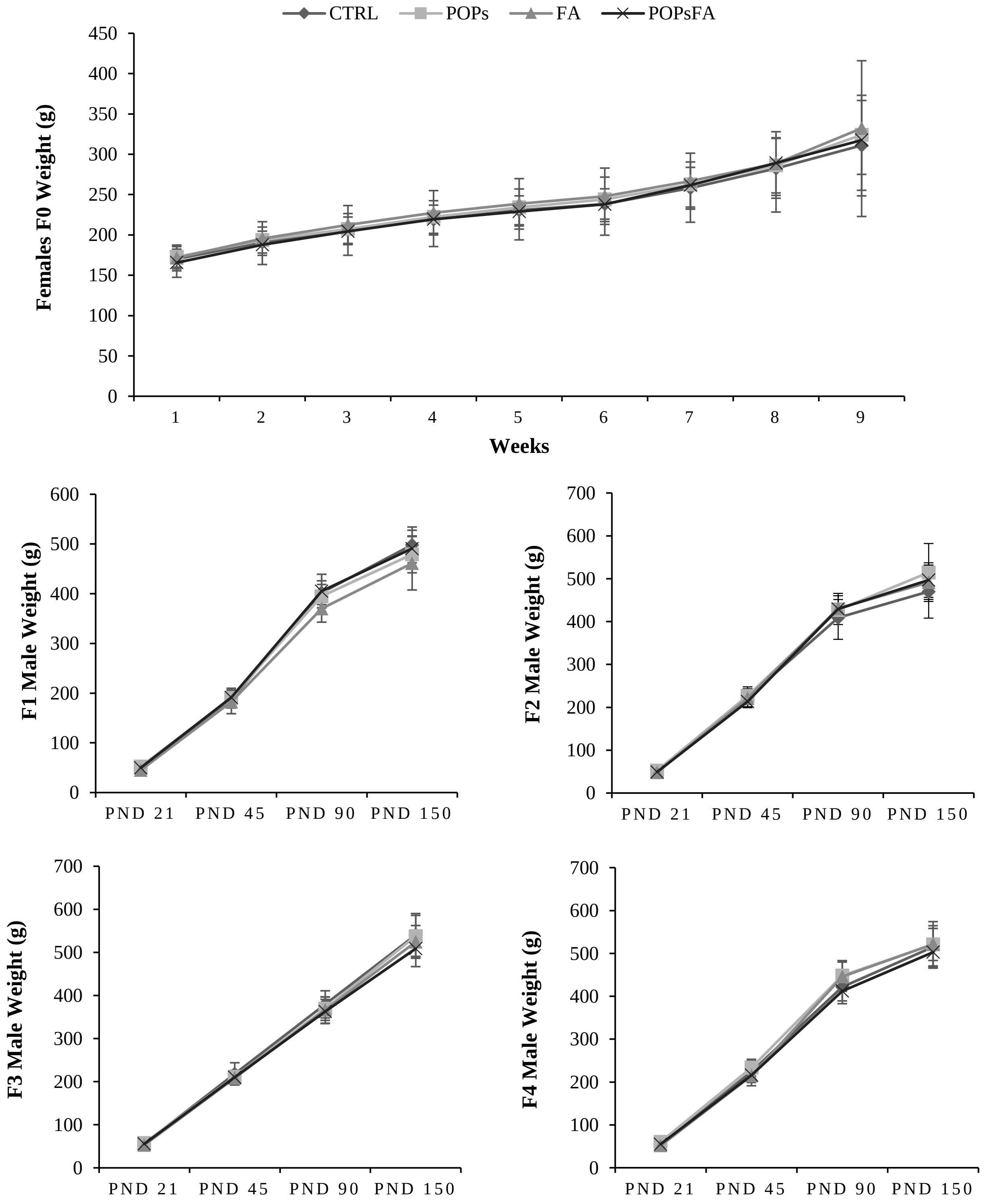

Supplement: Supplementary file 1 — Dataset 1 [file 41598_2019_50060_MOESM1_ESM.pdf]
